# Thermal and Combustion Properties of Biomass-Based Flame-Retardant Polyurethane Foams Containing P and N

**DOI:** 10.3390/ma17143473

**Published:** 2024-07-13

**Authors:** Jing Zhan, Liangchen Mao, Rongshui Qin, Jing Qian, Xiaowei Mu

**Affiliations:** 1 School of Civil Engineering, Anhui Jianzhu University, Hefei 230601, China; zhanjing@ahjzu.edu.cn (J.Z.); 17730482309@163.com (L.M.); 2 School of Environment and Energy Engineering, Anhui JianZhu University, Hefei 230601, China; anhuiqj2008@163.com; 3 State Key Laboratory of Fire Science, University of Science and Technology of China, Hefei 230026, China

**Keywords:** polyurethane foam, biomass, flame-retardant mechanism, char layer

## Abstract

Biomass has been widely used due to its environmental friendliness, sustainability, and low toxicity. In this study, aminophosphorylated cellulose (PNC), a biomass flame retardant containing phosphorus and nitrogen, was synthesized by esterification from cellulose and introduced into polyurethane to prepare flame-retardant rigid polyurethane foam. The combustion properties of the PU and PU/PNC composites were studied using the limiting oxygen index (LOI), UL-94, and cone calorimeter (CCT) methods. The thermal degradation behavior of the PU and PU/PNC composites was analyzed by thermogravimetric analysis (TGA) and thermogravimetric infrared spectroscopy (TG-IR). The char layer after combustion was characterized using SEM, Raman, and XPS. The experimental results showed that the introduction of PNC significantly improved the flame-retardant effect and safety of PU/PNC composites. Adding 15 wt% PNC to PU resulted in a vertical burning grade of V-0 and a limiting oxygen index of 23.5%. Compared to the pure sample, the residual char content of PU/PNC15 in a nitrogen atmosphere increased by 181%, and the total heat release (THR) decreased by 56.3%. A Raman analysis of the char layer after CCT combustion revealed that the ID/IG ratio of PU/PNC15 decreased from 4.11 to 3.61, indicating that the flame retardant could increase the stability of the char layer. The TG-IR results showed that PNC diluted the concentration of O_2_ and combustible gases by releasing inert gases such as CO_2_. These findings suggest that the developed PU/PNC composites have significant potential for real-world applications, particularly in industries requiring enhanced fire safety, such as construction, transportation, and electronics. The use of PNC provides an eco-friendly alternative to traditional flame retardants. This research paves the way for the development of safer, more sustainable, and environmentally friendly fire-resistant materials for a wide range of applications.

## 1. Introduction

Rigid polyurethane foam is widely used in building insulation, furniture, and the automotive industry due to its low thermal conductivity, excellent insulation properties, and superior mechanical performance. It holds significant potential, particularly as an external wall insulation material in construction [1,2]. However, polyurethane decomposes easily at high temperatures, releasing flammable gases and burning rapidly without proper flame-retardant measures. This combustion produces a large amount of heat and toxic smoke, posing a threat to human safety and health. Therefore, developing flame-retardant and smoke-suppressant polyurethane foam materials to slow the burning and thermal decomposition process during fires is crucial for protecting lives and property [3].

Halogen-free flame retardants are commonly added to rigid polyurethane foam to enhance its flame resistance. These retardants include metal hydroxides [4], organometallic frameworks [5], nanofillers [6], and phosphorus- or nitrogen-based compounds [7,8]. Typically, these retardants promote the formation of a char layer through chemical reactions. This char layer effectively insulates the material from oxygen and heat during combustion, thereby slowing the burning process [9,10]. Additionally, during the decomposition of polyurethane, halogen-free flame retardants can react with the decomposition products, reducing the formation of low molecular weight flammable gases and thus decreasing smoke release [11,12]. However, conventional halogen-free flame retardants have limitations such as low efficiency, high loading requirements, poor compatibility with polyurethane, significant environmental impact, and potential negative effects on the material’s processing performance and mechanical strength [13,14,15]. As a result, current research focuses on developing new, efficient, and environmentally friendly flame retardants and optimizing their compatibility with polyurethane.

Biomass flame retardants are compounds or products derived from biomass materials, and they are used to enhance a material’s resistance to flames and slow the burning process. These flame retardants typically come from renewable resources such as starch [16], cellulose [17], chitosan [18], and sodium alginate [19]. The use of biomass flame retardants aims to provide an environmentally friendly and sustainable way to improve the flame resistance of materials while reducing the environmental and health risks associated with traditional flame retardants, such as those containing halogenated compounds. Most biomass flame retardants contain hydroxyl groups, which can replace a portion of petroleum-based polyols in the synthesis of polyurethane, thereby reducing the use of non-renewable resources. Xing et al. [20] prepared a novel bio-based flame retardant—melamine starch phytate ester (PSTM)—by reacting phytic acid starch ester with melamine and introducing it into polyurethane foam. Their experimental results showed that PU foam with 30 wt% PSTM has a limiting oxygen index (LOI) value of 25.9% and a UL-94 rating of V-0. Chi J. et al. [21] synthesized phosphorus-containing soybean oil-based polyol (PSOP) and phosphorus polyol (HEDP-PO) using epoxidized soybean oil, 1-hydroxyethylidene-1,1-diphosphonic acid (HEDP), and propylene oxide (PO) as raw materials. These new polyols were used together to synthesize flame-retardant polyurethane foam (FRPUF), resulting in a reduction of the heat release rate from 395 kW/m^2^ to 251 kW/m^2^ and a total heat release from 24.9 MJ/m^2^ to 18.0 MJ/m^2^ compared to pure polyurethane samples. Song H. et al. [22] used adenosine triphosphate (ATP) and positively charged chitosan (CS) as a coating for combustible polymer material PU foam. Compared to pure samples, the peak heat release rate of ATP/CS-PU was reduced by 42.0%, and the total smoke release was significantly reduced by 30.6%. Zhang W. et al. [23] analyzed the synergistic flame-retardant performance of sodium alginate (SA), diethyl aluminum phosphite (ADPO2), and expandable graphite (EG) in polyurethane. The RPUF/5ADPO/7.5SA/7.5EG composition exhibited excellent thermodynamic, smoke suppression, and flame-retardant properties. The introduction of these biomass flame retardants significantly improves the flame resistance of materials, reduces the production of smoke and toxic gases, and enhances the fire safety of polyurethane insulation materials.

Currently, some research attempts have been made to use fossil- and petroleum-based materials to synthesize flame-retardant polyurethane foam, which is unsustainable. Moreover, most of the existing research on biomass-based flame retardants has focused on simple modifications, thus lacking a comprehensive exploration of their potential for high-performance flame retardancy. Therefore, in this study, cellulose was subjected to phosphorylation reaction to prepare a novel phosphorus–nitrogen–containing biomass flame retardant—aminophosphorylated cellulose (PNC)—which was followed by the preparation of PU/PNC composite materials. This approach leverages renewable resources, thus offering an eco-friendly solution while effectively enhancing PU foam’s flame retardancy. Unlike traditional flame retardants that often involve halogenated compounds, our approach minimizes environmental and health hazards. And the raw materials required for preparation are also not expensive, thus making it economically viable for widespread use in various industries. The thermal stability and combustion performance of PU and PU/PNC composite materials were characterized using TGA and CONE. The flame resistance and flame-retardant grades of PU and its composite materials were evaluated through LOI values and UL-94 tests. XPS, SEM, and Raman tests were used to characterize the char layer formed after the combustion of PU and its composite materials. Furthermore, TG-IR was employed to further study the generation of gas-phase products during the combustion process of polyurethane foam materials and to analyze their flame-retardant mechanism. By demonstrating the advantages of PNC, this study seeks to provide a sustainable and efficient alternative to conventional flame retardants, thus contributing to the development of safer and more environmentally friendly materials.

## 2. Experiments

### 2.1. Materials

Polyether polyol (LY-4110) is provided by Shandong Bluestar Dongda Co., Ltd. (Zibo, China). PAPI (PM-200) was supplied by China Wanhua Chemical Group Co., Ltd. (Yantai, China), and the silicon foam stabilizer (AK-8805) was obtained from Momentive High-tech Materials Co., Ltd. (Shanghai, China). TEA was purchased from Jining Huakai Resin Co., Ltd. (Jining, China). Triethylenediamine (A33, 33%) was obtained from Xuzhou Yihuiyang New Materials Co., Ltd. (Xuzhou, China). Dibutyltin dilaurate (LC) was supplied by Shanghai Aladdin Biochemical Technology Co., Ltd. (Shanghai, China). Cellulose, urea, and phosphite were purchased from Shanghai Macklin Biochemical Technology Co., Ltd. (Shanghai, China).

### 2.2. Preparation of PNC

**Figure 1:**
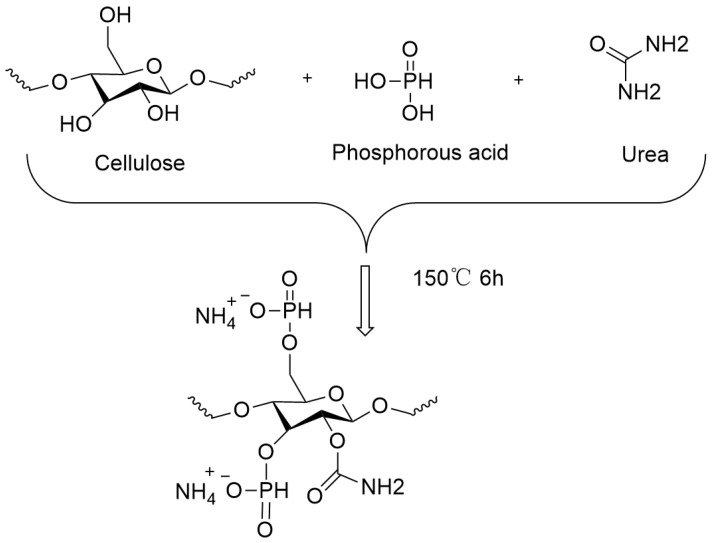
Preparation process of PNC.

In the first step, 312 g of urea was added to a 250 mL three-necked flask and heated to 140 °C under a nitrogen atmosphere, with vigorous stirring, until the urea melted. Then, 50 g of cellulose was added to the molten urea, followed by the addition of 257 g of hypophosphorous acid. Based on studies by Suflet D. M. et al. [24], the mixture was reacted at 150 °C for 6 h. The reaction mixture was dissolved in a 250 mL 1 N sodium hydroxide aqueous solution and precipitated using a solution of ethanol and water in a ratio of 1:2. This process was repeated three times to remove the urea and unreacted hypophosphite. Finally, the modified cellulose was dried at 70 °C for 4 h. A light yellow, water-soluble aminophosphorylated cellulose (PNC) was obtained. The phosphorylation synthesis process of the cellulose is shown in Figure 1. The yield of PNC was determined to be approximately 85%.

### 2.3. Preparation of Flame-Retardant Polyurethane

The raw materials required for the preparation of rigid polyurethane foam are listed in Table 1. Initially, in a foaming machine or mixing equipment, polyether polyol (LY-4110), triethylenediamine (A33), dibutyltin dilaurate (LC), deionized water, silicone oil (AK-8805), triethylamine (TEA), and PNC are mixed to form the polyol component. In the preparation process of PNC, the amount of each reagent is carefully adjusted to maintain the reaction mixture at a weakly acidic-to-neutral pH value, and a pH indicator is used to monitor the pH of the reaction mixture throughout the entire process. A small amount of ammonia solution is added as needed to adjust the pH within the desired range (approximately pH 5–7). It is essential to ensure thorough mixing and avoid air inclusion. The mixing was performed using a high-speed mechanical stirrer (IKA RW 20 digital, LC-ES-60, LICHEN, Shanghai, China) equipped with a three-blade propeller type stirrer. The stirring speed was set at 2000 rpm. Subsequently, the polyol components were rapidly mixed with polymer methylene diphenyl diisocyanate (PAPI) at room temperature (26 °C) for 2 min. The resulting mixture was immediately poured into a pre-prepared rectangular mold with internal dimensions of 150 mm (length) × 150 mm (width) × 50 mm (height). The foam was allowed to rise and cure at room temperature (60 °C) for 24 h. Post-curing, the foam was demolded and subjected to further curing in an oven at 60 °C for 12 h to ensure the complete crosslinking and stabilization of the PU foam.

### 2.4. Measurements and Characterization

The thermal properties of the samples were studied by using a cone calorimeter (Suzhou VOUCH Measurement Technology Co., Ltd., Suzhou, China). The sample size was 100 mm × 100 mm × 25 mm, and the thermal radiation power was 35 kW/m^2^.

The LOI was measured by an HC-2C oxygen index meter (Nanjing Jiangning Analytical Instrument Co., Ltd., Nanjing, China), and the sample size was 100 mm × 10 mm × 10 mm.

Polyurethane samples with dimensions of 127 mm × 13 mm × 10 mm were tested for vertical combustion grade according to ASTM D3801-2010 (Nanjing Jiangning Analytical Instrument Co., Ltd.).

The thermal stability of the sample increased from 30 °C to 800 °C at a heating rate of 20 °C/min on TGA (Germany Netzsch TG209, Selb, Germany) under an air and nitrogen atmosphere, respectively. The sample test amount was about 10 mg.

The morphology of the samples after CCT was studied using SEM (Hitachi SU8010, Kyoto, Japan). The samples were pre-treated by spraying gold. Small pieces of PU foam samples were cut to dimensions of approximately 5 mm × 5 mm × 3 mm to fit the SEM sample holder. The sputtering process was carried out using a Quorum Q150R Plus sputter coater (Quorum, Nanjing, China). Gold (Au) was chosen as the coating material.

TG-IR (PerkinElmer, Waltham, MA, USA) is a combination of a TGA8000 thermogravimetric analyzer and Frontier infrared spectrometer. The sample was placed in a crucible and heated from room temperature to 800 °C (in a nitrogen atmosphere and a flow rate of 30 cm^3^/min) by a heating rate of 20 °C/min. The wavenumber range of infrared measurement was 4000–600 cm^−1^, the background and sample scanning times were conducted 32 times, and the spectrum resolution was 4 cm^−1^.

The X-ray diffraction (XRD) data were obtained by using a TD-3500 device (Dandong Tongda Technology, Dandong, China), with a step size of 0.02° and a range from 10° to 80°.

X-ray photoelectron spectroscopy (XPS) was conducted with 10 kV Al Kα X-ray radiation on a Thermo Scientific K-Alpha instrument.

The instrument used for Raman spectroscopy was the SPEX-1403 laser pull spectrometer provided by SPEX Company of the United States, and the measurement range was from 500 to 2000 cm^−1^.

## 3. Results and Discussions

### 3.1. Chemical Properties of PNC

**Figure 2:**
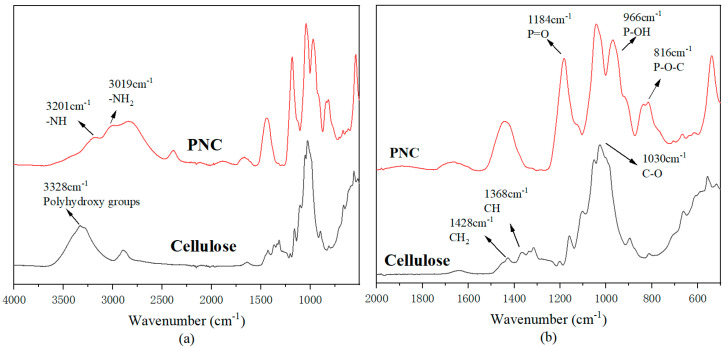
Infrared spectrum of the cellulose and PNC: (**a**) 4000–500 cm^−1^; (**b**) 2000–500 cm^−1^.

The infrared spectrum of cellulose before and after modification are shown in Figure 2a and Figure 2b, respectively. For cellulose, the main characteristic absorption peaks include the stretching vibration absorption peak of the C-O bond at 1030 cm^−1^; the stretching vibration peaks of the CH_2_ and CH bonds at 1428 cm^−1^ and 1368 cm^−1^, respectively; and the C-O bond from the glycoside unit or glycosidic bond appearing at 1030 cm^−1^. The peak at 3328 cm^−1^ corresponds to the stretching vibration of multiple hydroxyl groups. The absorption peak at 966 cm-1 in the PNC spectrum corresponds to the stretching vibration of P-OH [25], the peak at 1184 cm^−1^ corresponds to the P=O stretching vibration of the phosphate group [26], the peak at 2389 cm^−1^ corresponds to the P-H bond, and a band at 816 cm^−1^ corresponds to the P–O–C bonds [24]. The broad peak range of 3000–3300 cm^−1^ corresponds to -NH groups and -NH_2_ groups, indicating the involvement of urea in the synthesis reaction. In summary, based on the changes in the infrared spectrum, it can be confirmed that the target flame-retardant PNC was successfully synthesized.

**Figure 3:**
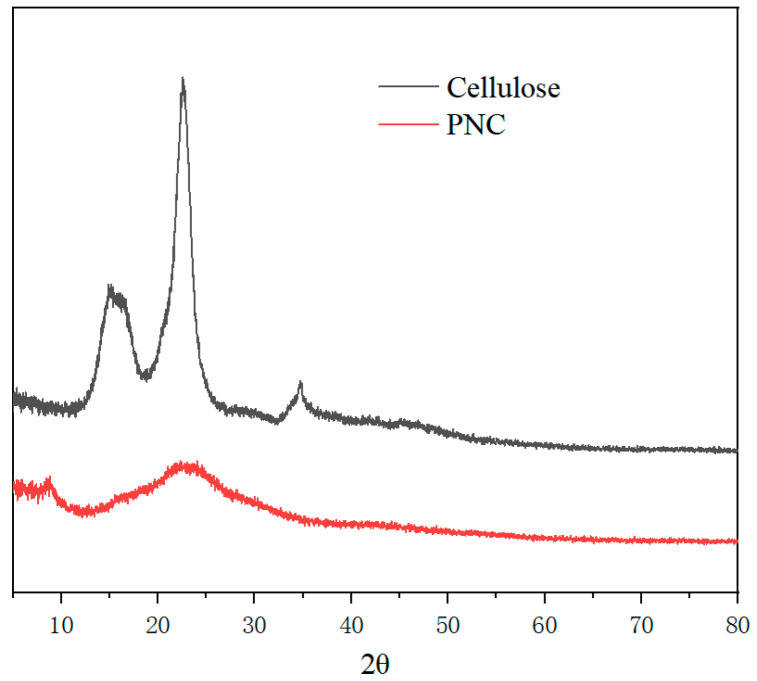
An XRD test diagram of the cellulose and PNC.

The effect of phosphorylation on the crystal structure of the cellulose was studied using an X-ray diffractometer (XRD). The XRD curves of cellulose and PNC are shown in Figure 3. The cellulose exhibited two sharp peaks around 16.5° and 22.8°, corresponding to the cellulose I crystal planes 200 and 110, which indicates its high degree of crystallinity and ordered structure. The crystallinity of MCC was calculated based on the empirical crystallinity index (CrI) [27].

(1) $$CrI=\frac{I_{200}-I_{am}}{I_{200}}\times 100\%$$

where *I*_200_ represents the intensity of the characteristic diffraction peaks of crystal planes, and *I_am_* indicates the intensity of non-crystalline diffraction (2θ = 18.6°). The crystallinity of cellulose was calculated to be 81.6%. In PNC, the intensity of the diffraction peaks weakened, and the peaks became broader and flatter, indicating that phosphorylation disrupts some of the cellulose’s crystal structures, thereby reducing its crystallinity. Phosphorylation leads to the breakage of hydrogen bonds between cellulose chains, reducing the number and size of ordered regions [28]. The reduction in crystallinity contributes to the enhancement of the material’s flame retardancy because non-crystalline regions are more likely to form stable char layers during thermal decomposition. The changes in the XRD spectrum of PNC samples are also direct evidence of chemical modification since phosphorylation leads to the formation of new amorphous forms.

**Figure 4:**
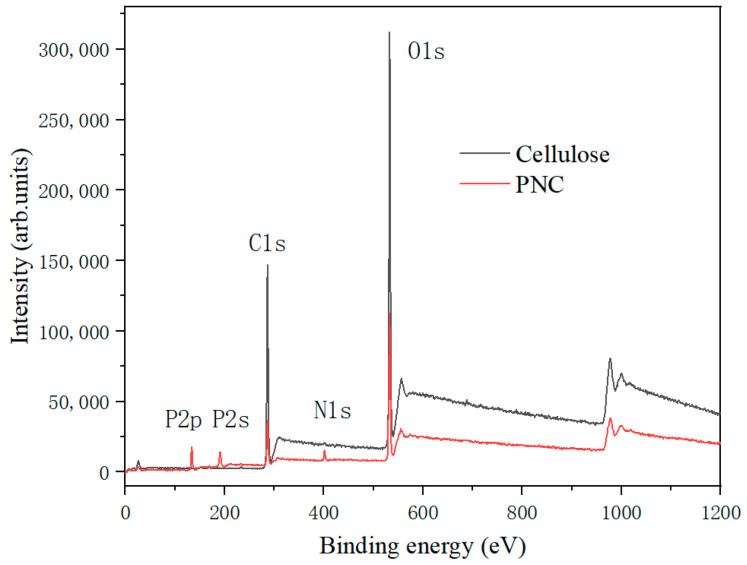
XPS spectra of the cellulose and PNC.

Figure 4 shows the XPS spectra of the PNC and cellulose. From the figure, it can be observed that PNC contains elements C, N, O, and P, indicating the successful incorporation of nitrogen and phosphorus into cellulose. The XPS curve of cellulose exhibited peaks of elemental char (C) and oxygen (O) at 530–536 eV and 282–298 eV, respectively, while the XPS curve of the PNC presented smaller peaks of P2p between 132 and 136 eV, which are not present in the natural cellulose spectrum. This directly confirms the presence of phosphorus, and it indicates that phosphorus has been successfully incorporated into the cellulose structure [25]. And a small peak near the binding energy of 403 eV appeared in the XPS curve of the phosphorylated cellulose, indicating the presence of a small amount of urea binding to the material surface during the phosphorylation modification process of cellulose [29], which also indicates the successful loading of element N onto the cellulose.

Meanwhile, changes in the elements before and after cellulose chemical modification can be observed through energy spectrometer testing. As shown in Table 2, it can be seen that, in cellulose, 60.3 wt% of char (C), 39.2 wt% of oxygen (O), and 0.5 wt% of nitrogen (N) elements were uniformly distributed on the surface. In addition to the char (C), oxygen (O), and nitrogen (N) elements, the PNC also contain 6.8 wt% of the phosphorus (P) element, which also proves that the phosphorus element had been successfully grafted onto the surface of the cellulose.

**Figure 5:**
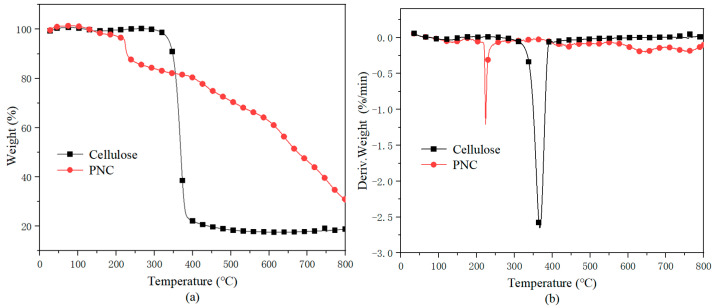
TGA curves of the cellulose and PNC in a nitrogen atmosphere. (**a**) TG; (**b**) DTG.

The thermal stability of the cellulose and PNC under a nitrogen atmosphere was studied using TGA, and the results are shown in Figure 5. From the graph, it can be observed that both cellulose and PNC exhibited three main thermal degradation stages. The first decomposition stage occurred between 100–200 °C, which was attributed to the evaporation of water and other volatile components. The second thermal degradation stage of cellulose occurred from 300 °C to 400 °C. The reason for the rapid decline in quality during this stage was that the cellulose molecules began to crack, and the β-glycoside ether bonds inside broke when heated, thus forming a large number of volatile substances such as levoglucan [30]. For the PNC, due to the catalytic degradation of phosphorus, this stage was reduced to around 223 °C. The third stage occurred at higher temperatures, typically between 400 and 800 °C, involving the further degradation of coke and the formation of stable residues. The maximum decomposition temperature (T_max_) of the cellulose was 367 °C, while that of PNC was reduced to 223 °C. Compared to cellulose, the onset decomposition temperature of the PNC decreased to 215 °C, and this was attributed to the catalytic degradation effect of the phosphorus. Phosphorus compounds promote early char formation, which facilitates the dehydration of cellulose and lowers the T_max_. Additionally, the residue at 800 °C for PNC was 31.7%, which is significantly higher than the unmodified cellulose residue of 11.3%. This is mainly due to the formation of acidic substances, such as phosphoric acid, during the decomposition process of PNC. These acids promote cross-linking and char formation, thereby reducing thermal decomposition and increasing the char residue [31].

### 3.2. Thermal Stability of the PU and PU/PNC Composite Materials

(1) Under a nitrogen atmosphere

**Figure 6:**
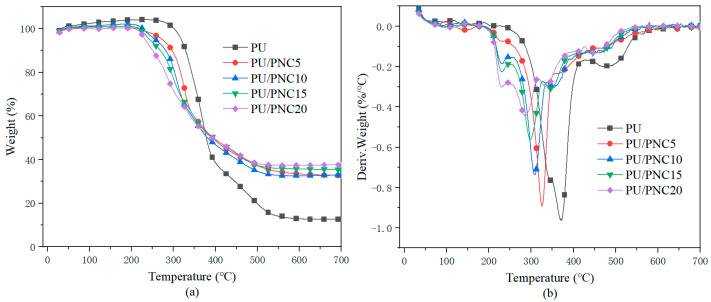
TGA curves of the PU and its composites under a nitrogen atmosphere. (**a**) TG; (**b**) DTG.

Figure 6 shows the TGA curves of the PU and PU/PNC composite materials, with detailed data presented in Table 3. From the graph, it can be observed that the pure polyurethane (PU) exhibited two distinct decomposition stages. The first stage occurred between 200–400 °C, where there was a rapid decrease in the sample mass due to the decomposition of urethane bonds (i.e., hard segments); the second stage occurred between 450–600 °C, corresponding to the decomposition of the soft segments, including polyols and isocyanates [32]. Upon introducing the biomass flame retardant into PU, a third thermal decomposition stage began, corresponding to the initial decomposition of the PNC. Compared to PU, the initial decomposition temperature (T_5%_, defined as the temperature at which 5% of the sample’s total mass decomposes) of PNC composite materials decreased. The flame-retardant mechanism of the PNC enhances the formation of a protective char layer at lower temperatures. This char layer acts as a barrier, slowing down the thermal degradation process and improving fire resistance. However, the early onset of char formation due to the catalytic action of phosphorus lowers the T_max_ of the composite, as the degradation process starts earlier [33]. The residue at 700 °C for PU/PNC5, PU/PNC10, PU/PNC15, and PU/PNC20 was 32.74%, 32.93%, 35.38%, and 37.54%, respectively. With the increase in flame-retardant content, the char residue was also on the rise, especially for PU/PNC20 as, compared to the pure sample’s char residue of 12.61%, it increased by 197%.

(2) Under an air atmosphere

**Figure 7:**
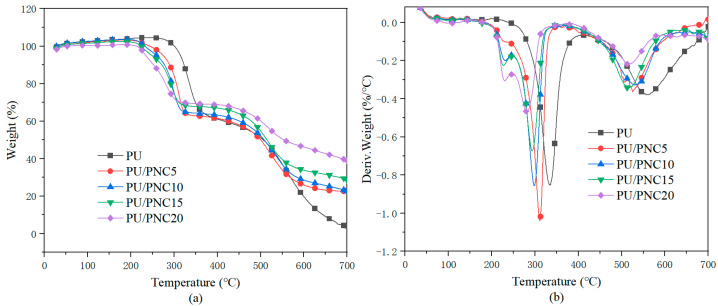
The TGA curves of PU and its composites under an air atmosphere. (**a**) TG; (**b**) DTG.

Thermogravimetric analysis (TGA) is a technique used to assess the thermal stability of materials by quantitatively and qualitatively analyzing the changes in mass at different temperatures. Figure 7 illustrates the TGA curves of the PU and its composite materials under an air atmosphere, with the detailed data provided in Table 4. From the graph, the T_5%_ for PU decreased from 318.8 °C to 232.9 °C for PU/PNC20, and similar trends were observed for T_max_. This decrease in these parameters indicated that the presence of PNC accelerated the initial thermal decomposition process of the PU matrix. As the flame-retardant content increased, the residue content also increased, especially for PU/PNC20, which showed a nearly tenfold increase compared to the 4.03% char residue of the pure sample. This indicated that the introduction of PNC enabled the formation of a protective char layer in polyurethane, thereby improving flame retardancy.

### 3.3. Combustion Properties of the PU and PU/PNC Composites

LOI and vertical burning tests are common methods used to assess the flame retardancy of materials, and the specific test results of this study shown in Table 5. The LOI value of PU was 19.4%, and the introduction of flame retardants was able to increase the material’s LOI value. Moreover, as the flame-retardant content increased, the LOI value continued to increase. The LOI value of PU/PNC15 was 23.5%, thus achieving a UL-94 V-0 rating. The reduction in the combustion length from 100 mm to 29 mm and the increase of the residual material from 2% to 35% after combustion indicated that PNC promotes the formation of a protective carbon layer, which serves as a barrier for heat and mass transfer. This barrier slows down the combustion process and reduces the degree of combustion.

**Figure 8:**
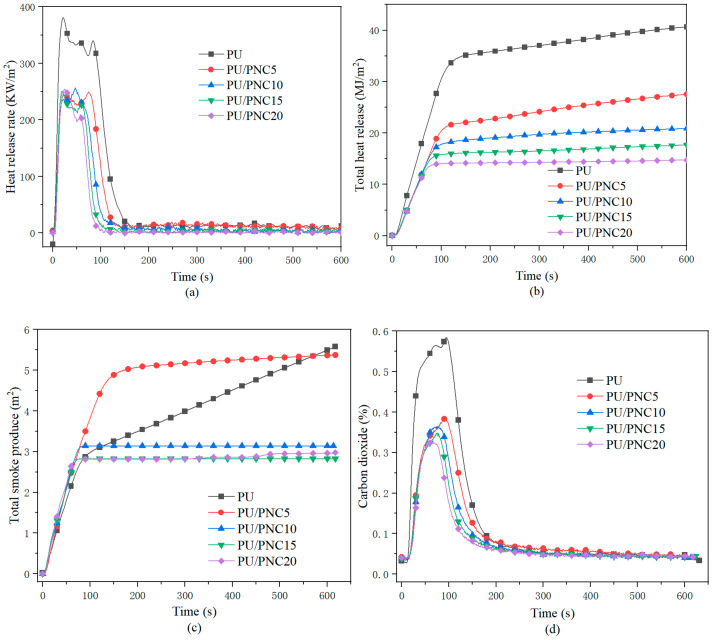
The CCT data of PU and its composites: (**a**) HRR; (**b**) THR; (**c**) TSP; and (**d**) CO_2_.

CCT was conducted on the PU and PU/PNC composite materials to investigate their combustion performance, as shown in Figure 5 and Table 6. The peak heat release rate (PHRR) of PU was 379.8 kW/m^2^, while that of PU/PNC15 was 251.2 kW/m^2^, which is a reduction of 33%. However, as the PNC content rose, the PHRR remained almost unchanged, possibly due to the slow formation of a uniform char layer, which only exhibited insulating properties after 100 s of combustion. The total heat release (THR) of PU was 40.8 MJ/m^2^, whereas that of PU/PNC15 decreased by 56.3%. From this, it can be inferred that the addition of cellulose phosphate makes the carbon layer that is formed on the surface of PU material denser during combustion, thus preventing the diffusion of heat from inside the material. The toxic smoke released during the combustion of polyurethane is usually harmful. The total smoke production (TSP) is a crucial parameter for studying a material’s smoke suppression performance, with smaller values indicating lower smoke concentration during combustion and thus less harm to personnel in case of fire. As shown in Figure 8c, the TSP of the PU, PU/PNC5, PU/PNC10, PU/PNC15, and PU/PNC20 composite materials was 5.58, 5.38, 3.14, 2.82, and 2.97 m^2^, respectively. The higher smoke production observed for the PU/PNC5 formulation was attributed to insufficient flame-retardant dispersion and incomplete char formation, thus leading to higher volatile compound release during combustion. The PU/PNC15 composite material reduced by 49.46% compared to PU alone, indicating the good smoke suppression effects of PNC. Moreover, when the flame-retardant content reached 15 wt%, the CO_2_ release decreased by 42.3% compared to the pure sample. In summary, the addition of flame-retardant PNC significantly reduced the heat release, smoke density, and CO_2_ generation of the polyurethane materials, thus greatly enhancing their fire safety [34].

### 3.4. Char Layer Analysis

**Figure 9:**
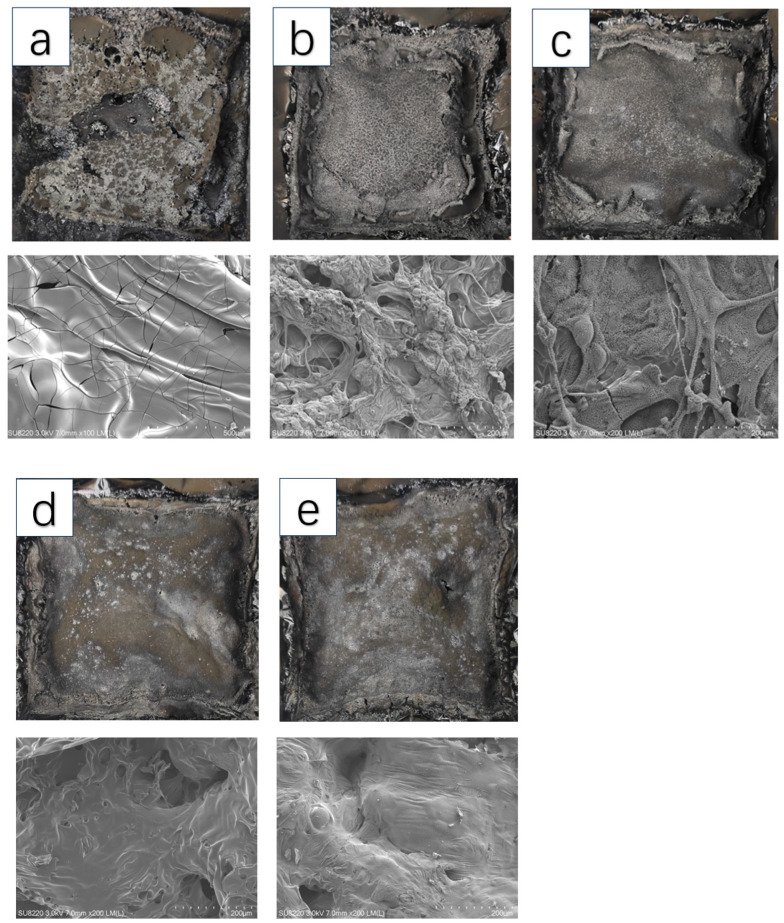
The macroscopic and microscopic morphologies of the char layer of PU and its composites after CONE test: (**a**) PU; (**b**) PU/PNC5; (**c**) PU/PNC10; (**d**) PU/PNC15; and (**e**) PU/PNC20.

The char layers of the PU and PU/PNC composite materials obtained through CCT are shown in Figure 9. The char layer of the PU appeared irregular and discontinuous, with minimal residual char after combustion, thus indicating a relatively weak char layer structure that was formed during PU combustion. The PU/PNC10 exhibited small pockmarks on the surface, which resulted from the release of volatile gases during combustion [35]. With an increase in PNC content, the white areas on the surface of the char layer also increase. These white areas were formed by the breakdown and decomposition of the phosphorus-containing fragments in the PU/PNC composite materials, such as phosphoric acid, pyrophosphate, and polyphosphate, which covered the surface of the char layer [36]. The char layer structures formed by PU/PNC15 and PU/PNC20 were very uniform and dense, effectively preventing heat and gas exchange. These char layers exhibited better thermal stability and protective effects.

**Figure 10:**
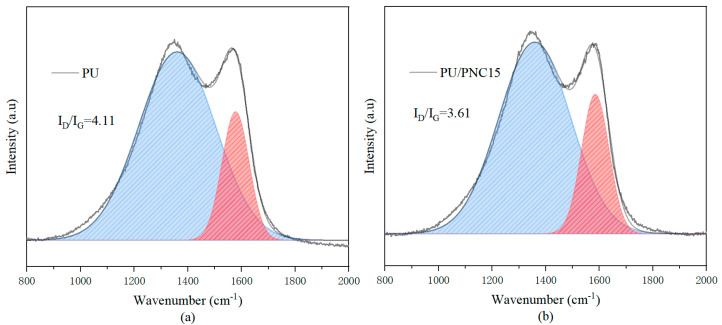
Raman fitting data for the residual carbon layers of PU and PU/PNC15. (**a**) PU; (**b**) PU/PNC15.

Raman spectroscopy was employed to study the graphitization degree of the residual char layers of the PU and PU/PNC15 after CCT. As shown in Figure 10, there were two peaks at 1565 cm^−1^ (G peak) and 1345 cm^−1^ (D peak), where the former represents the graphite structure, and the latter represents the amorphous carbon. The intensity ratio of the D peak to the G peak (ID/IG) indicated the degree of graphitization of the residual char layer. A smaller ID/IG ratio indicates a higher degree of graphitization of the char layer, thus allowing it to better protect the material and prevent further thermal degradation [37,38]. After the CONE test, the ID/IG value of the residual char layer of PU was 4.11, while that of PU/PNC15 was 3.61. This suggests that the graphitization degree of PU/PNC15 is higher than that of PU, thus further demonstrating that the synergistic effect of nitrogen and phosphorus benefits the formation of a denser char layer, thereby protecting the polyurethane foam effectively [36]. These findings highlight the effectiveness of PNC in enhancing the flame retardancy of PU composites. The ability of PNC to form a stable and protective char layer suggests that PU/PNC composites can be particularly useful in applications where high thermal stability and fire resistance are required, such as in construction materials, automotive components, and electronic housings.

### 3.5. Gas Phase Product Analysis

**Figure 11:**
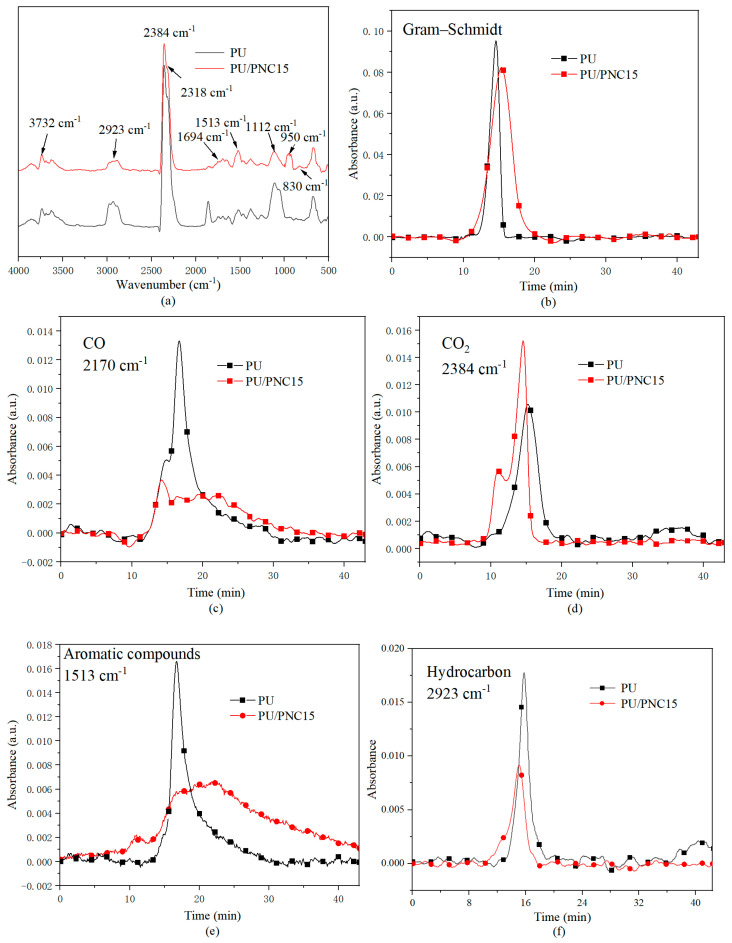
The TG-IR data of PU and its composites: (**a**) FTIR spectrum of the pyrolysis products of PU at a maximum decomposition rate, (**b**) Gram–Schmidt (GS) curves, (**c**) CO, (**d**) CO_2_, (**e**) aromatic compounds, and (**f**) hydrocarbon.

Thermogravimetric infrared spectroscopy (TG-IR) can be used for the quantitative analysis of gas products during sample pyrolysis processes [39]. Figure 11a corresponds to the infrared spectra associated with polyurethane and flame-retardant polyurethane in a nitrogen atmosphere. From the graph, it can be observed that the O-H bond of free water molecules appears at 3732 cm^−1^ [40], while the stretching vibration of carbon–hydrogen compounds occurs at 2923 cm^−1^. The characteristic peak of CO_2_ generated from decomposition appears at 2384 cm^−1^, and the NCO absorption peak is at 2318 cm^−1^, with the C=O bond appearing at 1850 cm^−1^ [41]. The P=O bond generates at 1112 cm^−1^, and the bending vibration of P-O-C occurs around 830 cm^−1^. A new peak is observed at 950 cm^−1^, indicating the production of ammonia (NH_3_) during the decomposition of PU/PNC15. The C-H (2923 cm^−1^) absorption intensity of PU/PNC15 is significantly reduced compared to PU, indicating that the formation of hydrocarbons and olefin products during pyrolysis is inhibited [42]. A new frequency band appears at 1694 cm^−1^, partly due to the formation of P-OH groups through the aforementioned chemical reactions. Meanwhile, the broad peaks of 1250 to 1130 cm^−1^ caused by the influence of hydrogen bonding on P=O indicate the formation of phosphate and polyphosphate [43].

Figure 11b illustrates the relationship curve between the total volatile gas intensity and time for PU and PU/PNC15. It is evident that PU/PNC15 exhibits a lower release of pyrolysis gas products, indicating that a stable char layer can suppress the decomposition of polyurethane and reduce the generation of gaseous products. Additionally, Figure 11c,e,f depict the absorption intensities of the CO, aromatic compounds, and hydrocarbons for PU and PU/PNC15, with PU/PNC15 showing significantly lower absorption intensities than PU. This was primarily attributed to the thermal decomposition products of PNC, such as polyphosphates, promoting the formation of char and consolidating more carbon in the condensed phase at high temperatures, thereby inhibiting the further decomposition of PU. Generally, aromatic compounds tend to aggregate into smoke particles [44], and higher concentrations of CO pose greater risks to individuals during fires, potentially causing coughing, asphyxiation, shortness of breath, and neurological discomfort upon inhalation of hydrocarbons. The reduction in hydrocarbons, aromatic compounds, and CO suggests fewer smoke particles and toxic gases formed during thermal degradation, which would be beneficial for firefighting and rescue operations during fire incidents [45]. Figure 11e demonstrates that the introduction of PNC leads to a significant increase in the release of CO_2_ during the pyrolysis process, and CO_2_, being inert, can dilute combustible gases, thereby preventing a further combustion of PU. From the above experimental results, it is evident that PU/PNC15 releases inert gases such as CO_2_ and during pyrolysis to dilute the concentrations of O_2_ and combustible gases in the gas phase [46]. The ability of PU/PNC composites to release inert gases and form a protective char layer enhances their fire resistance, thus making them suitable for use in high-risk environments such as construction materials, furniture, and electronic devices.

### 3.6. Flame-Retardant Mechanism

**Figure 12:**
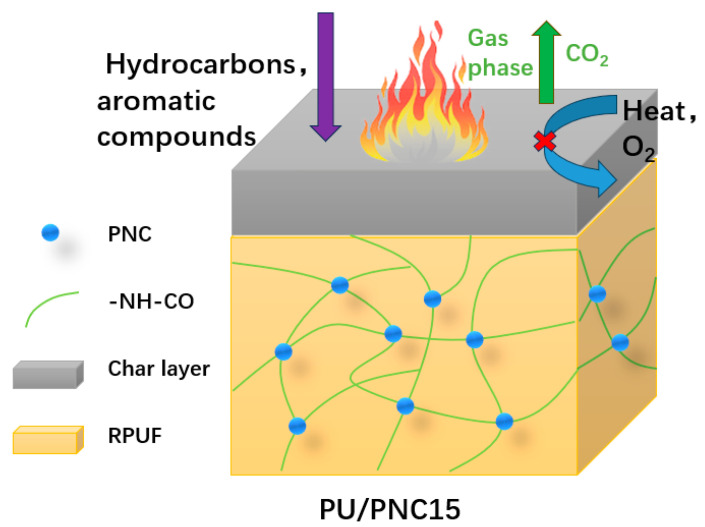
Schematic diagram of the flame-retardant mechanism of the PU/PNC15 composite.

PNC, as a phosphorus and nitrogen-containing biomass flame retardant, exhibits excellent flame-retardant properties. Figure 12 illustrates the potential flame-retardant mechanism of PU/PNC15 composite materials. By introducing phosphate ester groups and amino groups onto the surface of cellulose through phosphoric esterification reactions, it becomes a biomass flame retardant doped with P and N. The addition of PNC enables PU/PNC15 composite materials to achieve a V-0 rating in vertical burning tests. In the gas phase, the presence of PNC suppresses the thermal decomposition process of polyurethane materials, thereby reducing the release of volatile products such as hydrocarbons and aromatic compounds. The reduction in flammable small molecule products further weakens the combustion reaction. Simultaneously, inert gases such as CO_2_ are released during combustion to dilute the concentration of O_2_ and combustible gases in the gas phase, thereby inhibiting combustion. In the condensed phase, the breaking of P-O-C bonds and P=O bonds in phosphorus-containing segments of PU/PNC composite materials leads to the formation of phosphoric acid, pyrophosphates, and polyphosphates covering the surface of the char layer and acting as a barrier to restrict the release of O_2_ and heat. The generation of phosphoric acid further promotes the dehydration and carbonization of the matrix, increasing the residual char yield. In summary, the flame-retardant ability of PU/PNC composite materials is significantly enhanced through the combined action of the gas-phase and condensed-phase mechanisms.

## 4. Conclusions

This study synthesized a novel phosphorus and nitrogen-containing biomass flame-retardant, aminophosphorylated cellulose (PNC), and it incorporated it into PU to investigate the combustion and thermal properties of the prepared flame-retardant materials. The use of PNC as a flame retardant is novel, providing a sustainable and effective alternative to traditional flame retardants. The test results demonstrated that PU/PNC15 could achieve a UL-94V-0 rating. Thermal analysis results indicated that the presence of PNC significantly enhanced the thermal stability of PU/PNC composite materials at high temperatures. The residual char yield of the PU/PNC15 at 700 °C in a nitrogen atmosphere increased by 181%. The results from the cone calorimetry showed that the total heat release (THR) of PU/PNC15 was 56.3% lower than that of PU. In the gas phase, TG-IR testing revealed that the PU/PNC composite materials could reduce the release of CO during the pyrolysis process, while generating CO_2_ further dilutes the concentration of combustible gases and O_2_. In the condensed phase, PNC could catalyze the formation of a carbon network, increasing its degree of graphitization. These data indicate that PNC is an effective green flame retardant. The findings suggest that PU/PNC composites can be effectively used in high-risk applications such as construction materials, automotive components, and electronic housings due to their improved fire resistance. In the future, investigating the long-term stability and durability of PU/PNC composites under various environmental conditions would be valuable.

## Figures and Tables

**Table 1:** Formulations of PU and its composites.

Samples	LY-4110 (g)	PAPI (g)	A33 (g)	LC (g)	Water (g)	AK-8805 (g)	TEA (g)	PNC (g)
PU	100	150	1	0.5	2	2	3	0
PU/PNC5	100	150	1	0.5	2	2	3	13.60
PU/PNC10	100	150	1	0.5	2	2	3	28.72
PU/PNC15	100	150	1	0.5	2	2	3	45.62
PU/PNC20	100	150	1	0.5	2	2	3	64.63

**Table 2:** Element content of the cellulose and PNC.

Element	Cellulose (wt%)	PNC (wt%)
C	60.3	38.9
O	39.2	45.2
N	0.5	4.4
P	0	11.5

**Table 3:** Pyrolysis parameters of the PU and its composites under a nitrogen atmosphere.

Samples	T5% (°C)	Tmax (°C)			Char Residues at 700 °C (%)
		Step 1	Step 2	Step 3	
PU	318.8	372.6	483.6		12.61
PU/PNC5	273.9	230.7	325.7	375.9	32.74
PU/PNC10	256.9	231.5	309.1	354.1	32.93
PU/PNC15	243.5	228.7	299.1	343.5	35.38
PU/PNC20	232.9	230.2	284.3	332.7	37.54

**Table 4:** The pyrolysis parameters of PU and its composites under an air atmosphere.

Samples	T_5%_ (°C)	T_max_ (°C)			Char Residues at 700 °C (%)
		Step 1	Step 2	Step 3	
PU	314.6	333.7	567.7		4.03
PU/PNC5	274.6	225.1	311.6	525.9	23.21
PU/PNC10	259.6	231.8	294.8	540.9	22.71
PU/PNC15	248.3	226.1	290.9	518.2	28.59
PU/PNC20	234.3	229.5	280.1	520.4	39.01

**Table 5:** The UL-94 and LOI test results of the PU and its composites.

Samples	LOI	UL-94		Length Burnt (mm)	Residue Remaining (%)
		t_1_ + t_2_ (s)	Rating		
PU	19.4	>60	No rating	100	2
PU/PNC5	20.2	>60	No rating	74	11
PU/PNC10	21.6	16	V-1	61	14
PU/PNC15	23.5	9.4	V-0	53	22
PU/PNC20	24.6	6.3	V-0	29	35

**Table 6:** CCT data of the PU and its composites.

Samples	PHRR (KW/m^2^)	THR (MJ/m^2^)	TSP (m^2^)	CO_2_ (Kg/Kg)
PU	379.8	40.8	5.58	87.4
PU/PNC5	249.6	27.7	5.38	65.1
PU/PNC10	256.8	20.9	3.14	56.5
PU/PNC15	251.2	17.8	2.82	53.1
PU/PNC20	254.4	14.7	2.97	49.9

## Data Availability

The raw data supporting the conclusions of this article will be made available by the authors on request.
